# In Vivo Atlas of Neuroprotective Cyclic Dipeptides Derived from Food Gelatin Using Peptidomics and Feature-Based Molecular Networking

**DOI:** 10.1021/acs.jafc.5c08112

**Published:** 2025-10-22

**Authors:** Pingping Dong, Lanjia Ao, Yujie Li, Haoyuan Zeng, Yanmin Zhang, Haibo Zou, Jing Leng, Na Li, Jian-Lin Wu

**Affiliations:** † School of Pharmacy, Faculty of Medicine & State Key Laboratory of Quality Research in Chinese Medicines, 58816 Macau University of Science and Technology, Macau SAR 999078, China; ‡ Faculty of Chinese Medicine and State Key Laboratory of Quality Research in Chinese Medicines, 58816 Macau University of Science and Technology, Macau SAR 999078, China; § School of Pharmacy, Health Science Center, Xi’an Jiaotong University, Xi’an, Shanxi Province 710049, China; ∥ Guangxi Key Laboratory of Translational Medicine for Treating High-Incidence Infectious Diseases with Integrative Medicine, Department of Medical Immunology, 118330 Guangxi University of Chinese Medicine, Nanning, Guangxi Province 530200, China

**Keywords:** food gelatin, cyclic dipeptides, in vivo, peptidomics, Feature-based molecular networking, targeted spatiotemporal metabolomics

## Abstract

Food gelatin is widely used in the food industry, yet its *in vivo* active substances remain unclear. This study proposes a strategy for *in vivo* research on donkey-hide gelatin that integrates peptidomics, feature-based molecular networks (FBMN), and targeted spatiotemporal metabolomics to address the above issues. Peptidomics based on database searching revealed a time-dependent decrease in peptide numbers and an increase in oligopeptide ratios in the small intestine over 24 h, with oligopeptides increasing from 50 to 100%. FBMN visualization in serum allowed the first identification of cyclodipeptides (CDPs) originating from gelatin. Targeted metabolomics detected 35 CDPs in serum, with 12 proline/hydroxyproline-CDPs being particularly abundant. Spatiotemporal analysis revealed different distributions of CDPs in the gastrointestinal and serum, and they showed prolonged retention *in vivo*. Twelve proline/hydroxyproline-CDPs displayed neuroprotective effects in HT-22 cells, with cyclo­(Hyp-Ala) showing the highest efficacy. This study presents a strategy for identifying exogenous bioactives in complex matrices, applicable to other food proteins.

## Graphical abstract

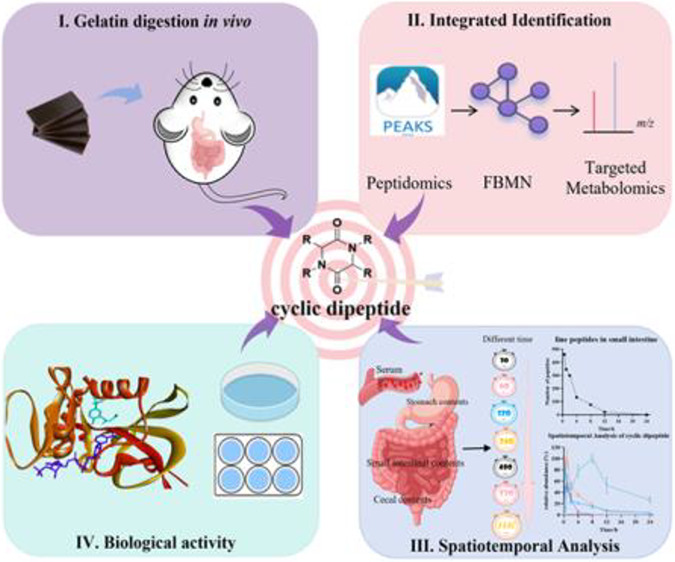

## 1 Introduction

Food gelatin, a protein-based hydrocolloid derived from partially denatured collagen, is widely used for its structural stability and nutritional properties.^1^ Collagen accounts for about 30% of mammalian protein,^2^ with type I collagen, composed of Gly-Xaa-Yaa repeats often containing proline (Pro) and 4-hydroxyproline (4-Hyp), being the most abundant.^3^ Donkey-hide gelatin (DHG) is a solid gelatin made from the dried or fresh skin of the equid donkey (*Equus asinus* L.) by decoction, concentration, and other processes. The pharmacological effects of DHG mainly include immune regulation,^4^ antioxidants,^5^ and neuroprotection.^6^ The main components of DHG are proteins from donkey skin.^6,7^ Previous research on gelatin hydrolysates has predominantly focused on the absorption, distribution, and bioactivity of linear peptides (e.g., the characteristic dipeptide Pro-Hyp and tripeptide Gly-Pro-Hyp),^3,8^ the fate of another significant class of peptides generated during gelatin processing and digestion, cyclic dipeptides (CDPs), remains poorly understood. CDP is formed by two amino acids connected into a stable diketopiperazine ring structure. It has been reported that X-Hyp-Gly can be easily converted into a Hyp-containing CDP by heating,^3^ which is an inevitable product of collagen/gelatin degradation. Increasing evidence highlights that CDPs possess diverse and significant bioactivities themselves, including antimicrobial, antiviral, immunomodulatory, signaling regulatory, and potential neuromodulatory effects.^9^ However, since DHG is predominantly administered orally, the specific bioactive compounds generated through *in vivo* digestion and metabolism, along with their dynamic transformation processes, remain to be fully elucidated.

Due to the presence of a diverse range of endogenous enzymes, peptides, and amino acids in the gastrointestinal tract and blood, with some peptides even being highly concentrated, a complex matrix effect arises. After digestion *in vivo*, exogenous proteins are converted into shorter peptides with lower abundance, which are difficult to distinguish from complex endogenous peptides. This phenomenon makes it extremely difficult to study and analyze the protein digestion process and active substances. In order to identify exogenous bioactive substances in a complex *in vivo* matrix, a common method is to use stable isotope labeling to track exogenous peptides *in vivo*. This method adds isotope-labeled amino acids or proteins to the diet to distinguish exogenous peptides from endogenous peptides.^10^ However, stable isotope labeling also has limitations, such as the complex and expensive synthesis and labeling processes of stable isotope labeling. Peptidomics is a primary approach for peptide analysis. By database searching, it can identify protein-specific peptides and reduce endogenous interference. However, current search engine scoring systems struggle to reliably identify short peptides (2–5 amino acids).^11^ Feature-based molecular networking (FBMN) is a powerful tool for visualizing compound abundance and detecting smaller peptides containing 2–5 amino acids. By using relative peak areas from input data, FBMN generates pie charts that display compound distribution across samples, and comparison between the blank and administration groups enables the rapid identification of exogenous compounds in a complex matrix.^12^

To overcome these challenges and efficiently identify bioactives from DHG in complex biological matrices, we combined peptidomics with FBMN analysis to efficiently identify *in vivo* bioactives from DHG in complex biological matrices. Peptidomics analysis, using the database search function, allowed us to specifically target peptides derived from DHG, while FBMN provided the ability to detect smaller peptides (2–5 amino acids) and visualize compound distribution. Furthermore, targeted spatiotemporal metabolomics was employed to comprehensively explore active substances from DHG, enabling the identification of key bioactives in serum with a high abundance and stability. The workflow of this study is shown in Figure 1.

**Figure 1:**
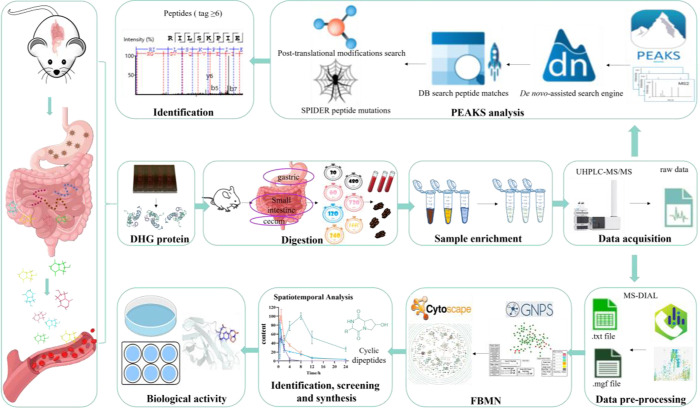
Workflow of the comprehensive analysis in the digestion of gelatin protein.

## 2 Materials and Methods

### 2.1 Materials

Five batches of DHG (batch numbers: 2307011, 2308019, 2308008, 2306022, and 2306006) samples were collected from Dong’e Co., Ltd. (Shandong, China). Anaqua Chemicals Supply (Hong Kong, China) provided HPLC-grade acetonitrile (ACN) and methanol (MEOH). Milli-Q water was prepared by using a Millipore water purification system (Billerica, MA, USA). All other chemicals used were analytical-grade. Synthetic CDPs (purity >95%) were ordered from ROYO biotech Co., Ltd. (Shanghai, China).

### 2.2 DHG Administration and Digestion Sample Preparation

Forty-five C57 mice (6–8 weeks, male) were obtained from SPF (Beijing) biotechnology Co. Ltd. The mice were maintained on a 12-h light/12-h dark cycle in a controlled environment with standard temperature and humidity levels of 24 ± 2 °C and 70 ± 5%, respectively. Following a week of acclimatization, the mice were divided into eight groups at random: the DHG groups (0.17, 0.5, 1, 2, 4, 8, 12, and 24 h after oral administration, *n* = 5) for test serum and contents in gastric, small intestine, and cecum and the control group (0 h, *n* = 5) for blank serum and digestive tract contents. The mice were fasted for 12 h with free water access before the experiment. Mice in the DHG groups were orally given a dose of 1000 mg/kg of body weight. Blood samples (1 mL) were taken from the suborbital venous plexus of mice at 0-, 0.17-, 0.5-, 1-, 2-, 4-, 8-, 12-, and 24-h postadministration. To separate the serum samples, each sample was centrifuged for 10 min at 3500 rpm. After blood was collected, mice were sacrificed, and the contents of the gastric, small intestine, and cecum were collected. The Macau University of Science and Technology’s institutional Animal Care and Use Committee authorized the animal protocols. The Guide for the Care and Use of Laboratory Animals (USA National Research Council, 1996) was complied with by the animal facilities and protocols.

To each 200 μL serum sample was added 600 μL of methanol and centrifuged at 3500 rpm for 10 min at 4 °C. The supernatant was dried using N_2_. Digestive tract contents samples (10 mg) were ultrasonically extracted with 75% methanol (1 mL) for 15 min, and the resulting supernatant was dried at room temperature using N_2_. The residue was reconstituted with 100 μL of 75% ACN (containing 0.1% TFA) for LC-MS/MS analysis.

### 2.3 Peptidomics Analysis

LC-MS/MS was performed on an Agilent 6546 LC/Q-TOF mass spectrometer. The flow rate was set to 0.3 mL/min. Solution A (0.1% formic acid in water) and Solution B (0.1% formic acid in ACN) comprised the mobile phase. Elution was performed using the following gradient: 0–3 min, 2–6% B; 3–10 min, 6–35% B; 10–27.9 min, 35–95% B; 27.9–28.0 min, 95–2% B. An ACQUITY UPLC BEH C18 column (2.1 × 100 mm, 1.7 μm, Waters, Milford, CT, USA) was used to perform the separation. The operating parameters in positive ion mode were as follows: vaporizer temperature, 350 °C; included charge states, 1–4. The mass spectrometer scanning range was *m*/*z* 150–2250, and the injection volume was 1 μL.

### 2.4 PEAKS Studio Database Searching

Database search engines are critical in peptidomics studies because they submit raw MS/MS spectra into data containing protein IDs.^13^ PEAKS Studio was employed for Peptidomic analysis in this work, resulting in sensitive and accurate peptide identification. Raw data were imported into PEAKS and compared to the Uniport *Equus asinus* database, using mass tolerances of 10 ppm for precursor ions and 0.02 Da for fragments. The cleavage enzymes were pepsin and trypsin, which allowed up to two missing cleavages. Deamidation (+0.98), oxidation (+15.99), and acetylation (+42.01) were designated as variable modifications. An average local confidence (ALC) score of ≥50% was used for the database search. Furthermore, the PEAKS software managed to identify unexplained PTM and common mutations using Peaks PTM, as well as additional mutations using SPIDER.

### 2.5 Feature-Based Molecular Networking

The raw data of LC-MS were converted into.mzML files using the MSConvert software. Then, the files were processed in MS-DIAL 4.90 with reference to the previous parameters of our team.^14,15^ The results were exported as TXT and MGF files and uploaded to the GNPS platform for analysis. The FBMN parameter settings were also slightly modified with reference to the previous parameters.^15,16^ The molecular network results were downloaded from the GNPS job status pages (https://gnps.ucsd.edu/ProteoSAFe/status.jsp?task=f90fc486a6184489a728f90b85991cfb, https://gnps.ucsd.edu/ProteoSAFe/status.jsp?task=0d3b6d4e9f6d40e3ab89014407304937), and visualized in Cytoscape 3.6.1.

### 2.6 Targeted Spatiotemporal Metabolomics Analysis

A targeted approach was used to run aliquots of samples on an Agilent 6550 LC/Q-TOF mass spectrometer to analyze the CDPs. For quantification in DHG, the standards of 12 CDPs were dissolved in 75% methanol (v/v) to prepare calibration solutions. Twelve calibration levels were prepared in 75% methanol at 5, 10, 50, 100, 200, 500, 1000, 1500, 2000, 5000, 10,000, and 20,000 ng/mL. For each of the five batches, 1 mg of DHG powder was weighed into a 1.5 mL tube and extracted with 75% methanol by ultrasonication for 30 min at room temperature. The extract was centrifuged (13,500 rpm, 10 min), and the supernatant was transferred to vials for LC–MS injection. Quantitation was performed by integrating peak areas and applying a 12-point weighted calibration curve (*R* ^2^ ≥ 0.999). The limits of quantification (LOQs) were determined based on signal-to-noise ratios of 10.

For gastrointestinal content analysis, freeze-dried powders (1 mg each) of stomach, small intestine, and cecum contents collected over the 0–24 h postadministration period were accurately weighed. The samples were extracted using the same pretreatment procedure as applied for DHG, namely, ultrasonication with 75% methanol for 30 min at room temperature, followed by centrifugation (13,500 rpm, 10 min). The supernatants were collected, dried under nitrogen, and reconstituted in 75% methanol for LC–MS analysis. Quantitation was performed against the 12-point calibration curves established above, ensuring consistency with the DHG quantitation workflow. Samples from different time points were analyzed individually to characterize the temporal distribution of CDPs within each gastrointestinal compartment.

For pharmacokinetic analysis, CDPs in serum were quantified using a matrix-matched calibration approach. Blank serum was spiked with CDP working solutions to prepare calibration standards (19.531–10,000 ng/L, 10 levels). Calibrators were processed in parallel with the study samples to account for matrix effects and extraction losses. Serum samples collected after DHG administration were treated with three volumes of cold methanol for protein precipitation, followed by centrifugation (13,500 rpm, 10 min). The supernatants were dried under nitrogen, reconstituted in 75% methanol, and analyzed by LC–MS under the same conditions as those for DHG quantitation. Pharmacokinetic parameters, including *C*_max_, *T*_max_, and AUC_0–24h_ were calculated using noncompartmental analysis with DAS 2.0 software.

### 2.7 Cell Culture and Drug Treatment

HT-22 cells, a mouse hippocampus neuronal cell line, are commonly used as a neuroprotective model cell.^17^ Glutamate (Glu) plays a crucial role in rapid excitatory synaptic transmission and normal physiological functions of the central nervous system. However, elevated concentrations of Glu in the intercellular space can induce oxidative stress and neurotoxicity, resulting in neuronal degeneration, aging, and cell death. This Glu-induced neurotoxicity is a significant mechanism contributing to neuronal cell death in neurodegenerative diseases.^18^ Hydrogen peroxide (H_2_O_2_) is one of the main substances produced by oxidative stress, and H_2_O_2_ has been considered as a therapeutic target for treating oxidative stress related to neurodegenerative diseases.^19^ In this experiment, two nerve injury models were established using Glu-induced HT-22 cells and H_2_O_2_-induced HT-22 cells aimed at investigating the protective effects of the tested CDP, thereby inferring its neuroprotective activity.^20^

HT-22 cells were cultured in DMEM medium with 10% fetal bovine serum (Gibco), 1% penicillin, and streptomycin (Gibco) at 37 °C. HT-22 cells were seeded at a density of 1 × 10^4^ cells per well in 96-well plates and cultured in DMEM medium containing 10% fetal bovine serum and 1% penicillin–streptomycin at 37 °C with 5% CO_2_. To establish the injury model, cells were exposed to a range of Glu (0–40 mM) or hydrogen peroxide (H_2_O_2_, 0–800 μM) concentrations for 24 h. Cell viability was determined by CCK-8 assay, and the concentration that resulted in 40–60% survival was selected as the modeling concentration. Accordingly, 20 mM Glu and 400 μM H_2_O_2_ were chosen for subsequent experiments.

For neuroprotection assays, cells were pretreated with CDPs (0, 25, and 50 μM) for 4 h, followed by cotreatment with Glu (20 mM) or H_2_O_2_ (400 μM) for an additional 24 h. Vehicle-treated cells without Glu or H_2_O_2_ served as the control group, and cells treated with Glu or H_2_O_2_ alone were used as the model group. Cell viability was assessed by measuring the absorbance at 450 nm using the CCK-8 assay. The results were further analyzed to evaluate the dose–response relationship of the CDP treatment.

### 2.8 Molecular Docking Simulation

Molecular docking was used to estimate the interactions between the CDPs and the target Kelch ECH-associating protein 1 (Keap-1) protein. The X-ray crystallographic structure of Keap-1 (PDB ID: 6ZF4) was acquired from the Protein Data Bank (http://www.rcsb.org/pdb). The 2D structures of CDPs were drawn by ChemDraw and converted to 3D structures using the ChemDraw 3D software. The docking procedures involved the elimination of the ligand and crystallographic water molecules from the protein, the addition of hydrogens, and the correction of the protein’s chemistry for absent atoms, constraints, and interactions. The molecular structures of CDPs and proteins were transformed to the PDBQT format for docking calculations using Auto Dock Tools. The rotatable bonds of the ligand remain free, and the receptor remains rigid and is assigned hydrogen atoms and Gasteiger charges. Auto Dock was used for molecular docking, and the model with the lowest free energy was selected for visual analysis.

### 2.9 Data Analysis

GraphPad Prism version 8.0.2 was used for statistical analysis, and the data are presented as the mean ± standard error. Differences among multiple groups were analyzed by one-way analysis of variance (ANOVA) followed by Tukey’s post hoc test. Comparisons between two groups were conducted using Student’s *t-*test where appropriate, and differences were considered significant when *p* < 0.05.

## 3 Results and Discussion

### 3.1 Database-Searching-Based Peptidomics Analysis of Long Peptides

The main component of DHG is protein, among which collagen is the most abundant. It is usually considered that the food-derived proteins are gradually digested into peptides, amino acids, and their derivatives.^6^ Therefore, we employed peptidomics technology to analyze the metabolites of DHG proteins *in vivo*. Given that the intestine is the primary site for digestion and absorption, the small intestine contents were first analyzed by using peptidomics. To eliminate the interference of endogenous peptides in mice, the database (DB) search function of PEAKS Studio was applied to identify unique peptides derived from donkey proteins as follows. Downloaded the FASTA file of *Equus africanus asinus* protein sequences from UniProt, and imported it into PEAKS software to construct a local protein library for the donkey species. The MS/MS data from small intestinal contents at various time points (0.5, 1, 2, 4, 8, 12, and 24 h) postadministration of DHG were imported into PEAKS Studio for analysis. The following post-translational modifications (PTMs) were considered: deamidation (+0.98), oxidation (+15.99), and acetylation (+42.01). Our results (Table 1, S1–S6) revealed a time-dependent decrease in the number of peptides identified in the small intestine, with 460 peptides detected at 0.5 h, and a progressive reduction to 0 peptides at 24 h. This observation suggested a dynamic degradation of DHG proteins over time. Further analysis of the peptide size distribution showed that at 0.5 h, oligopeptides (6–10 amino acids) and polypeptides (>10 amino acids) were present in equal proportions (50% each). However, as time progressed, we observed a marked shift: polypeptides decreased, while oligopeptides increased. At the 12-h mark, all identified peptides were oligopeptides, indicating a gradual breakdown of DHG proteins into progressively smaller peptide fragments.

**Table 1:** Results of the Number and Length of Peptides in Small Intestinal Contents at Different Times

time/h	number of peptides	oligopeptides	polypeptide
0.5	460	50.00%	50.00%
1	347	63.89%	47.26%
2	299	72.58%	27.42%
4	133	81.95%	18.05%
8	76	81.58%	18.42%
12	20	100.00%	
24	0		

Concurrently, to investigate the active peptides from DHG that enter the bloodstream, we also performed peptidomics analysis on serum samples. Our analysis did not detect donkey-derived peptides with a length of more than 5 amino acids in the serum, likely because DHG protein was gradually degraded into short peptides (<6 amino acids) before absorption, but these short peptides are not effectively identified by a database-dependent searching approach.

### 3.2 FBMN Analysis of Short Peptides

The database search engine algorithm identifies peptides via the comparison of acquired MS/MS spectra with those in the database; consequently, it relies heavily on the identification of peptides with more than 6 amino acids, which constrains the discovery of shorter bioactive peptides (2–5 amino acids). Therefore, to detect small peptides and exclude the influence of complex matrix effects, we used FBMN to rapidly analyze MS/MS data and find DHG-related metabolites. Based on the similarity of mass spectrometric signals, FBMN aligns experimental spectra using the MS-Cluster method. It organizes structurally related molecules with strong spectral similarity into distinct groups by recognizing common fragment ion patterns. Importantly, FBMN can visually represent the abundance of compounds at each time point in the form of a pie chart based on the relative peak area, enabling the rapid identification of exogenous compounds with zero abundance at the 0 h time point in large datasets.

The total FBMN of intestinal contents and serum samples is shown in Figure 2A,B, where each node represents a feature molecule. The molecular weight of the nodes is indicated by a color scale, ranging from green (150 Da) to yellow (690 Da, average molecular weight of 5 peptides) to red (1714 Da). Our focus was on peptides of 2–5 amino acids, so we concentrated on the green and yellow nodes. We used FBMN to generate a pie chart for the peptides smaller than 690 Da (Figure S1, Table S7), where black represents compounds in the 0-h (control) matrix and other colors represent peptides detected at different times following oral administration of DHG. As shown in Figure S1, two large clusters lack black nodes, indicating that these peptides are absent from the small intestinal matrix. The representative small molecule peptide nodes are shown in Figure 2C: one cluster consists of CDPs, while the other is composed of dipeptides. We compared the relative amounts of these peptides in the small intestine over a 4 h period and found that most dipeptides were detected only within the first 1 h, whereas CDPs were still detectable at 4 h. Similarly, Figure 2D shows the peptides identified in the serum, which were mainly dipeptides and CDPs. Some CDPs, such as cyclo­(Phe-Hyp), cyclo­(Val-Pro), cyclo­(Hyp-Leu), cyclo­(Leu-Phe), and cyclo­(Leu-Pro), were absent in the blood matrix and were detectable within 0.5–4 h (Figure 2D). Five dipeptides were also detected, namely Asp-Phe, PyroGlu-Phe, PyroGlu-Ile, Glu-Phe, and Phe-Leu. The first three existed for a short time and were only detected in the first hour, while the last two existed for a long time and could still be detected at 4 h. By comparing the proportions of the pie charts, it was found that although the levels of these dipeptides increased after taking DHG, they were also present in the blank serum (Figure 2D). Combining the peptidomics with the FBMN results, we were drawn to the CDPs that were not originally present in the *in vivo* matrix.

**Figure 2:**
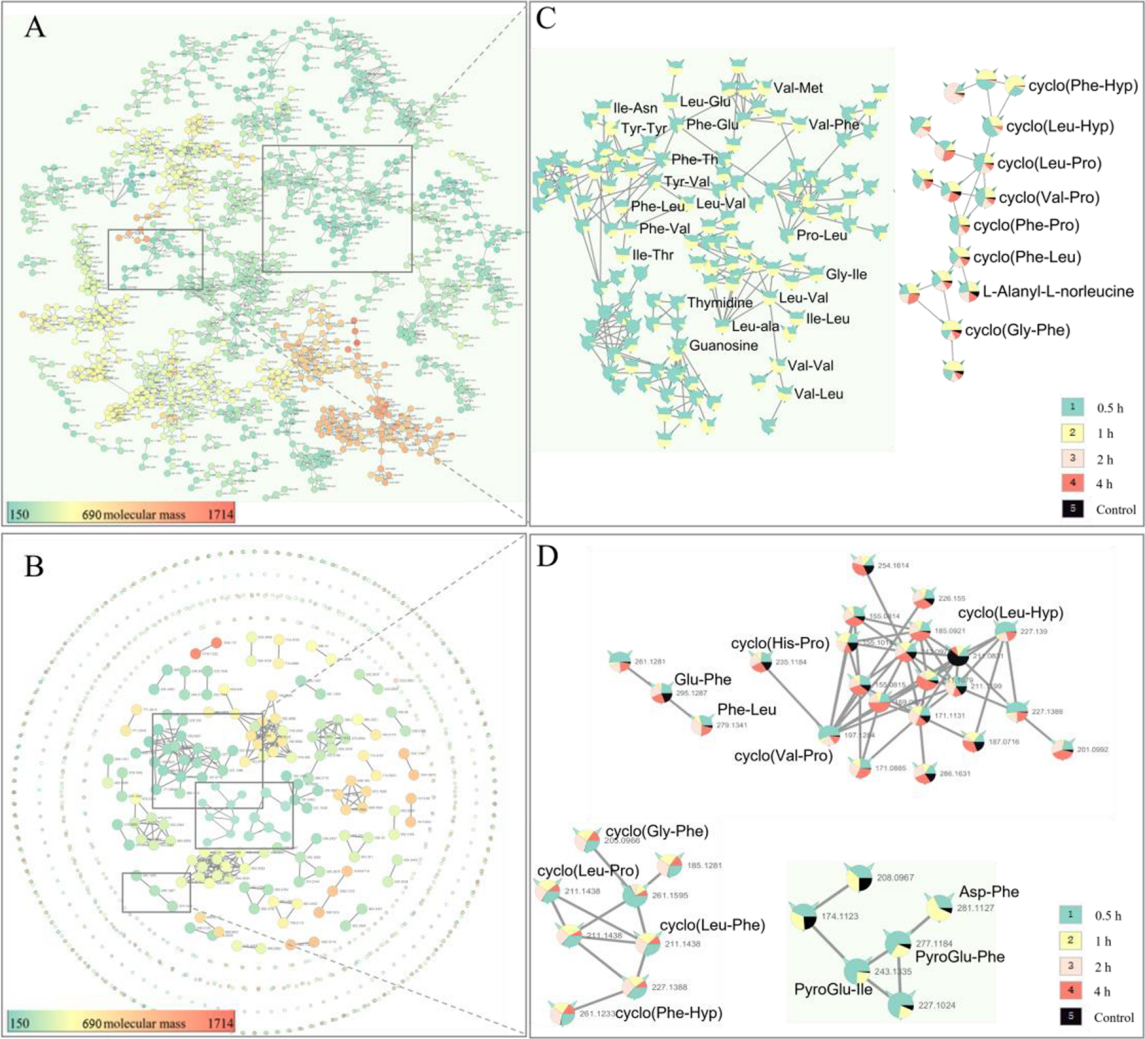
FBMN results of peptides in the small intestine and serum. (A, B) Total FBMN of small intestine and serum samples with a cosine similarity score cutoff of 0.7. (C, D) The molecular network of CDPs and dipeptides in the small intestine and serum. The black color in the pie chart represents the content of the compound at the 0 h time point, the green color represents the content at 0.5 h, the yellow color corresponds to 1 h, the pink color represents 2 h, and the red color corresponds to 4 h.

### 3.3 Targeted Metabolomics Analysis of Cyclic Dipeptides

In light of the above results, CDPs could be effectively transferred from the intestine to the bloodstream and have been reported to have multiple biological activities, such as anticancer, anti-inflammatory, and neuroprotective effects.^21^ However, data preprocessing is required before FBMN runs, which may result in the exclusion of some low-abundance metabolites in serum. Therefore, we established a CDP library for targeted metabolomics analysis to effectively search for more CDPs entering the serum. Since collagen does not contain tryptophan and cysteine residues,^22,23^ the remaining 18 amino acids were combined into CDPs in pairs, and their molecular formulas were calculated to establish the database (Table S8). Due to the high content of Hyp in collagen, cyclo­(Hyp-Xaa) derivatives were also included in the library to improve the identification of relevant peptides. The results showed that a total of 35 CDPs were searched in the serum (Table S9). Among them, 12 contained Pro or Hyp, and their intensities were also relatively high. Both Pro and Hyp are key amino acids of collagen and possess unique structural characteristics, such as the pyrrolidine ring in proline and the hydroxylation of proline in hydroxyproline, which contribute to their stability and role in collagen’s triple helix structure, making them important candidates for further investigation.

To further verify the structures of Pro/Hyp-CDPs, we synthesized the corresponding standards and conducted LC–MS/MS analysis. Figure 3A showed the chromatogram of the CDP standards in the positive ion mode, and Table 2 summarized their retention time (RT) and MS/MS fragment ions. By comparing the RT and MS/MS spectra of the samples and reference, we identified these Pro/Hyp-CDPs (Figures 3B and S2). Taking cyclo­(Gly-Pro) and cyclo­(Gly-Hyp) as examples, mass spectrometry fragmentation analysis revealed the unique fragmentation rules of CDPs. cyclo­(Gly-Pro) generated its [M+H]^+^ ion at *m*/*z* 155.0819 (C_7_H_11_N_2_O_2_) with the retention time of 1.90 min. In the ESI-MS/MS spectrum (Figure 3C), the precursor ion lost CO and 2 CO to form ions at *m*/*z* 127.0868 ([M+H-27.99 Da]^+^) and *m*/*z* 99.0917 ([M+H-55.99 Da]^+^), successively. In addition, it produced a Pro residue ion at *m*/*z* 98.0600, and it lost a CO to generate an ion at *m*/*z* 70.0615. As shown in Figure 3D, cyclo­(Gly-Hyp) yielded two significant product ions at *m*/*z* 143.0817 ([M+H-27.99 Da]^+^) and *m*/*z* 115.0817 ([M+H-55.99 Da]^+^), which indicated the continuous neutral loss of CO from the [M+H]^+^ ion at *m*/*z* 171.0766 (C_7_H_11_N_2_O_3_). Besides, it generated the product ion at *m*/*z* 153.0672 ([M+H-18.01 Da]^+^) by loss of one H_2_O. The Hyp residue ion was generated at *m*/*z* 114.0554 ([C_5_H_8_NO_2_]^+^), which then generated the ion at *m*/*z* 86.0603 by loss of CO. Significantly, the two carbonyl groups in the CDPs caused two consecutive neutral losses of CO (−27.99 and −55.99 Da), which serve as diagnostic ions for their identification (Table 2).

**Figure 3:**
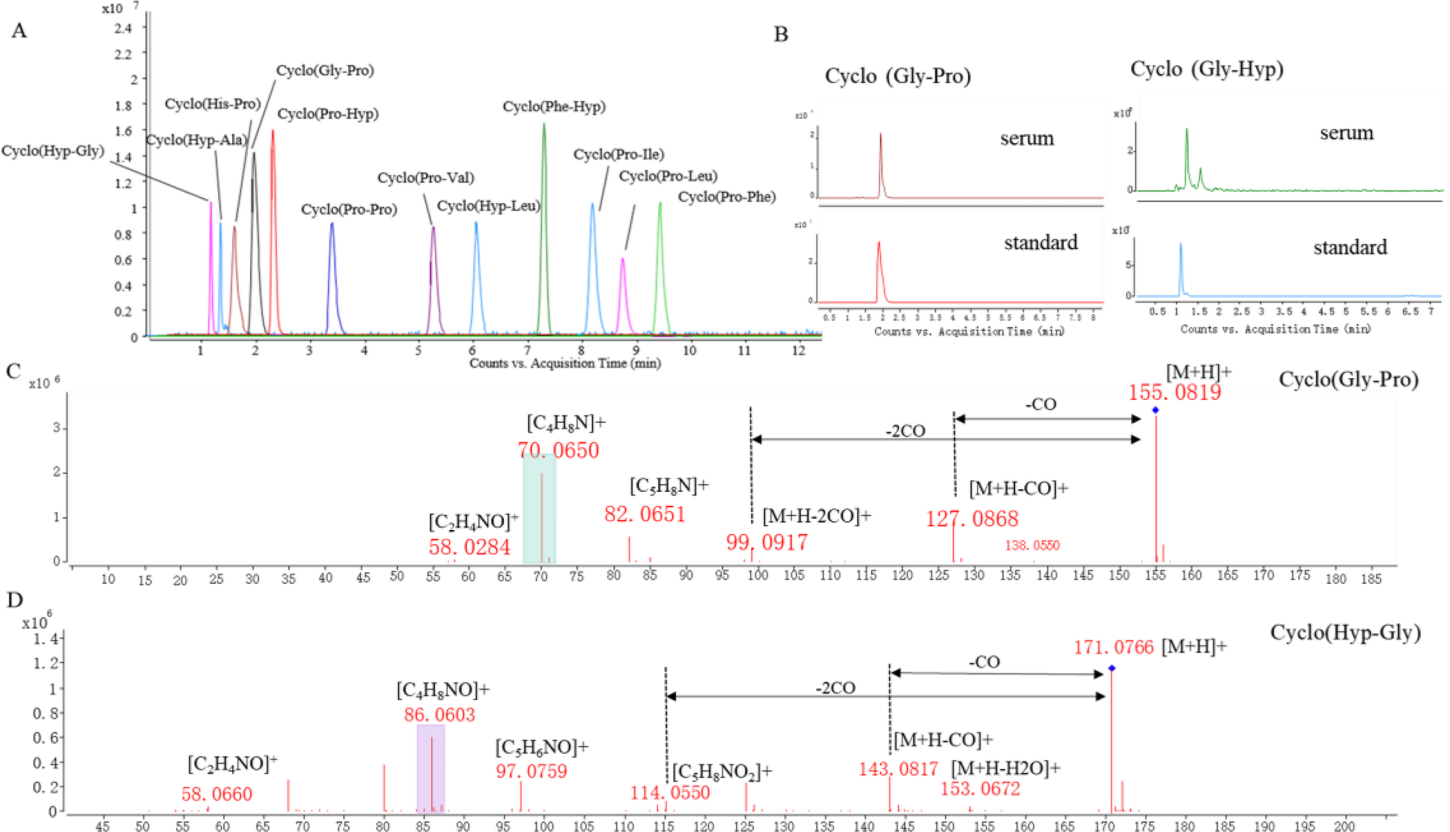
(A) LC–MS chromatogram of cyclic dipeptide standards. (B) LC–MS chromatograms of cyclo­(Gly-Pro) and cyclo­(Gly-Hyp) in serum (top) and standard solution (bottom). The MS_2_ spectra of cyclo­(Gly-Pro) (C) and cyclo­(Gly-Hyp) (D).

**Table 2:** Summary of CDPs Containing Pro and Hyp from DHG^a^

no.	compound	formula	mass	*m*/*z*	RT	MS/MS fragment ions	NLFs
1	cyclo(Gly-Pro)	C_7_H_10_N_2_O_2_	154.0737	155.0815	1.9	127.09 (−CO), 99.09 (−2CO), 98.06 (C_5_H_8_NO), 82.07 (C_5_H_8_N), 70.07 (C_4_H_8_N), 58.03 (C_2_H_4_NO)	27.99 (CO), 55.99 (2CO)
2	cyclo(Gly-Hyp)	C_7_H_10_N_2_O_3_	170.0686	171.0764	1.2	154.05 (−OH), 153.08 (-H_2_O), 143.08 (−CO), 115.09(−2CO), 96.04 (C_5_H_6_NO), 80.05 (C_5_H_6_N)	18.01 (H_2_O), 27.99 (CO), 55.99 (2CO)
3	cyclo(Hyp-Ala)	C_8_H_12_N_2_O_3_	184.0842	185.0921	1.4	168.07, 167.08 (−H_2_O),157.10 (−CO), 139.09 (−H_2_O–CO), 129.11(−2CO), 114.05 (C_5_H_8_NO_2_), 86.06 (C_4_H_8_NO), 72.04, 70.07 (C_4_H_8_N)	18.01 (H_2_O), 27.995 (CO), 46.00 (H_2_O+CO), 55.99 (2CO)
4	cyclo(Pro-Hyp)	C_10_H_14_N_2_O_3_	210.0999	211.1077	2.3	194.10, 193.09 (−H_2_O), 183.12 (−CO), 165.11 (−H_2_O–CO), 155.13(−2CO), 114.06 (C_5_H_8_NO_2_), 98.06 (C_5_H_8_NO), 86.06 (C_4_H_8_NO), 70.07 (C_4_H_8_N)	18.02 (H_2_O), 27.99 (CO), 46.00 (H_2_O+CO), 55.99 (2CO)
5	cyclo(Pro-Phe)	C_14_H_16_N_2_O_2_	244.1206	245.1285	9.4	217.13 (−CO), 189.14 (−2CO), 120.08, 98.06 (C_5_H_8_NO), 70.07 (C_4_H_8_N)	27.99 (CO), 55.99 (2CO)
6	cyclo(Pro-Pro)	C_10_H_14_N_2_O_2_	194.1049	195.1128	3.4	167.08 (−CO), 139.09 (−2CO), 98.06 (C_5_H_8_NO), 84.08 (C_5_H_10_N), 82.06 (C_5_H_8_N), 70.06 (C_4_H_8_N)	28.03 (−CO), 55.98 (−2CO)
7	cyclo(Phe-Hyp)	C_14_H_16_N_2_O_3_	260.1161	261.1234	7.2	243.11 (-H_2_O), 233.13 (−CO), 215.12 (-H_2_O–CO), 205.14(−2CO), 187.12 (-H_2_O-2CO), 170.10, 120.08, 114.05 (C_5_H_8_NO_2_), 86.06 (C_4_H_8_NO), 70.06 (C_4_H_8_N), 68.05 (C_4_H_6_N)	18.01 (H_2_O), 27.99 (CO), 46.00 (H_2_O+CO), 55.99 (2CO), 74.00 (H_2_O+2CO)
8	cyclo(Pro-Val)	C_10_H_16_N_2_O_2_	196.1212	197.1285	5.1	169.13 (−CO), 154.07, 141.14 (−2CO), 124.11 (C_8_H_14_N), 100.08 (C_5_H_10_NO), 98.06 (C_5_H_8_NO), 84.08 (C_5_H_10_N), 72.08 (C_4_H_10_N), 70.06 (C_4_H_8_N)	27.99 (CO), 55.99 (2CO)
9	cyclo(His-Pro)	C_11_H_14_N_4_O_2_	234.1111	235.119	1.5	207.12 (−CO), 179.10 (−2CO), 166.06 (C_7_H_8_N_3_O_2_), 110.07, 82.05 (C_5_H_8_N), 70.07 (C_4_H_8_N)	27.99 (CO), 56.02 (2CO)
10	cyclo(Hyp-Leu)	C_11_H_18_N_2_O_3_	226.1317	227.139	6.2	209.17 (-H_2_O), 199.14 (−CO), 171.15 (−2CO), 114.05 (C_5_H_8_NO_2_), 100.11, 86.06 (C_4_H_8_NO), 80.05 (C_5_H_6_N), 70.07 (C_4_H_8_N), 68.05 (C_4_H_6_N)	17.98 (H_2_O), 27.99 (CO), 55.99 (2CO)
11	cyclo(Pro-Leu)	C_11_H_18_N_2_O_2_	210.1363	211.1441	8.67	194.1177(−OH), 183.1490(−CO), 155.1541(−2CO), 98.0913(C_5_H_8_NO+), 70.0650(C_4_H_8_N+)	27.99(CO), 55.99(2CO)
12	cyclo(Pro-Ile)	C_11_H_18_N_2_O_2_	210.1363	211.1441	8.12	194.1177(−OH), 183.1490(−CO), 155.1541(−2CO), 98.0913(C_5_H_8_NO+), 70.0650(C_4_H_8_N+)	27.99(CO), 55.99(2CO)

a RT: retention time; NLFs: neutral loss fragments.

### 3.4 The Spatiotemporal Metabolomics Analysis of CDPs Containing Pro/Hyp

Furthermore, the spatiotemporal dynamic changes of Pro/Hyp-CDPs in vivo were systematically profiled across gastrointestinal tract compartments and blood circulation. The concentrations of CDPs in gastric, small intestine, cecal contents, and serum samples collected at 0, 0.17, 0.5, 1, 2, 4, 8, 12, and 24 h were analyzed using our established targeted metabolomics approach. The compartment-specific kinetic profiles were mapped, as illustrated in Figure 4. Furthermore, we quantified CDPs in five independent batches of DHG to estimate the dosage of each CDP and calculate the AUC_0–24_ upon administration. Table S10 presents the linear range and LOQ of the established quantification method, demonstrating its high sensitivity. Table 3 summarizes the CDP contents in five DHG batches as well as their pharmacokinetic parameters.

**Figure 4:**
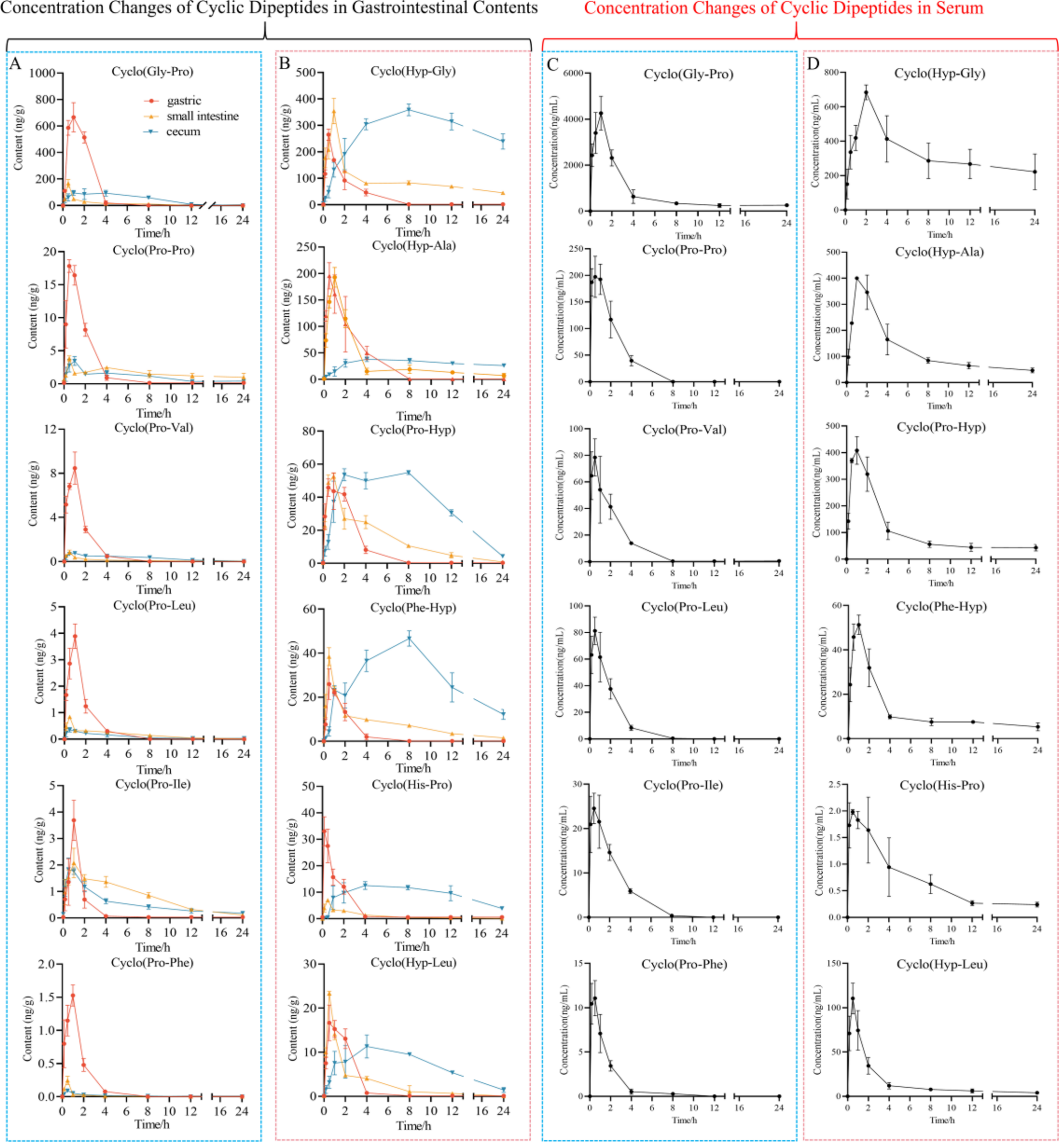
Concentrations of cyclic dipeptide contents in the stomach, small intestine, cecum, and serum after oral administration of DHG within 24 h (0, 0.17, 0.5, 1, 2, 4, 8, 12, 24 h). (A, B) Concentration changes of CDPs in gastrointestinal contents. (C, D) Concentration changes of CDPs in serum. Red denotes the stomach, yellow represents the small intestine, blue denotes the cecum, and black represents the serum. The data represent the mean ± SD.

**Table 3:** Contents of Cyclodipeptides in DHG and Pharmacokinetic Parameters of Cyclodipeptides Identified in Serum

compound	content in DHG (mg/g)						dosage (mg/kg)	*C*_max_ (μg/L)	*T*_max_ (h)	AUC_0–24h_ (μg/L h)
	DHG-1	DHG-2	DHG-3	DHG-4	DHG-5	mean ± SD				
cyclo(Gly-Pro)	1.5397	1.7825	1.506	1.6256	1.5068	1.5921 ± 0.1171	14.9260	4259.0300	1	15547.0170
cyclo(Hyp-Gly)	0.8883	1.0108	0.9118	0.9886	0.9119	0.9423 ± 0.0539	8.8340	683.7471	2	7371.6951
cyclo(Hyp-Ala)	0.5462	0.6139	0.5185	0.5838	0.4985	0.5522 ± 0.0471	5.1770	400.1572	1	2561.8131
cyclo(Pro-Hyp)	0.1221	0.1402	0.1175	0.1301	0.1199	0.1260 ± 0.0093	1.1810	408.1371	1	2126.6252
cyclo(Pro-Pro)	0.0517	0.0556	0.0528	0.0541	0.0536	0.0536 ± 0.0014	0.5020	197.3334	0.5	487.9963
cyclo(Hyp-Leu)	0.0384	0.046	0.0370	0.0410	0.0364	0.0397 ± 0.0039	0.3730	110.4803	0.5	301.1580
cyclo(Phe-Hyp)	0.0300	0.0347	0.0292	0.0322	0.0278	0.0308 ± 0.0027	0.2890	51.2931	1	263.2271
cyclo(Pro-Val)	0.0175	0.0205	0.0171	0.0209	0.0170	0.0186 ± 0.0019	0.1740	78.4401	0.5	201.4294
cyclo(Pro-Leu)	0.0065	0.007	0.0068	0.0071	0.005	0.0065 ± 0.0009	0.0610	81.2502	0.5	176.6962
cyclo(Pro-Ile)	0.0033	0.0035	0.0034	0.0037	0.0036	0.0035 ± 0.0001	0.0032	24.5331	0.5	71.9933
cyclo(Pro-Phe)	0.0027	0.0028	0.0027	0.0027	0.0028	0.0027 ± 0.0001	0.0025	11.0670	0.5	19.8164
cyclo(Pro-His)	0.0327	0.0349	0.0349	0.0384	0.0352	0.0352 ± 0.0020	0.0330	1.9830	0.5	13.9981

It is well-known that the small intestine is the main site of digestion and absorption, so we first compared the contents of CDPs in the small intestine and serum. After DHG ingestion, the CDPs content in the small intestine increased rapidly, and most of them reached the *T*_max_ at 0.5–1 h (Figure 4A,B). Subsequently, the content in serum also increased to *T*_max_ at 0.5–2 h after ingestion (Figure 4C,D). In serum, cyclo­(Gly-Pro) showed the highest systemic exposure (*C*_max_ = 4259.030 μg/L, AUC_0–24_ = 15547.017 μg/L·h), followed by cyclo­(Hyp-Gly) (*C*_max_ = 683.747 μg, AUC_0–24_ = 7371.695 μg/L·h), indicating rapid absorption and high bioavailability. In DHG, cyclo­(Gly-Pro) was also the most abundant CDP (1.5921 ± 0.1171 mg/g), followed by cyclo­(Hyp-Gly) (0.9423 ± 0.0539 mg/g). Consistent with the Gly-Pro-Hyp repeats in collagen,^1^ these results demonstrate a strong concordance between the compositional abundance of CDPs in DHG and their systemic exposure in vivo. In the stomach, CDP concentrations peaked during the early stage (0–2 h) after administration and then declined rapidly, becoming nearly undetectable after 4 h. Interestingly, high concentrations of six Pro-CDPs were observed in the stomach during this short period (Figure 4A), far exceeding their levels in the small intestine and cecum. This suggests that these CDPs may be absorbed in the stomach, rapidly taken up in the upper small intestine, or further metabolized during gastrointestinal transit. Peptide transporter 1 (PEPT1), which facilitates the uptake of peptides across the epithelial membrane in the small intestine, has also been reported to be expressed in the stomach,^24,25^ supporting the possibility of gastric absorption. Additional experiments are needed to distinguish between these possibilities.

Furthermore, we observed differences in the elimination rates of Pro- and Hyp-CDPs in the serum. As for absorption behavior, most Pro-CDPs (Figure 4C) peaked rapidly (*T*_max_ 0.5 h) and declined within 8 h, whereas Hyp-CDPs (Figure 4D) peaked more slowly (*T*_max_ 0.5–2 h) and persisted up to 24 h. For example, cyclo­(Pro-Hyp) remained detectable at 24 h, while cyclo­(Pro-Pro) returned to the baseline by 8 h. Similar trends were observed for cyclo­(Phe-Hyp) vs cyclo­(Phe-Pro) and cyclo­(Gly-Hyp) vs cyclo­(Gly-Pro), suggesting slower elimination of Hyp-CDPs. Interestingly, as shown in Figure 4B, we found that six CDPs exhibited progressive accumulation in the cecum, and in some cases, such as cyclo­(Hyp-Gly), cyclo­(Pro-Hyp), and cyclo­(Phe-Hyp), their concentrations at 8 h even surpassed the peak levels observed in the stomach and small intestine. The concentration of cyclo­(Hyp-Gly) reached 358.966 ng/g in the cecum at 8 h, which was comparable to or even exceeded its levels in the small intestine at 1 h (354.578 ng/g) and in the stomach at 0.5 h (265.229 ng/g). This suggests that these CDPs may either accumulate in the cecum due to incomplete absorption or be produced locally, thereby maintaining detectable concentrations in circulation for up to 8 h. Previous studies have reported that in the presence of peptone, *Lactobacillus reuteri* can produce up to 53 different CDPs, and lactic acid bacteria have also been shown to synthesize CDPs,^26,27^ providing supportive evidence for our hypothesis. However, further experimental verification is needed; for example, using germ-free or antibiotic-treated models will help verify whether gut microbiota can indeed generate CDPs and distinguish DHG-derived from microbiota-derived CDPs.

In addition, the elimination times of linear dipeptides and CDPs of the same amino acid composition were also compared. It was reported that the linear dipeptide Gly-Pro rapidly absorbed to a peak and decreased to a negligible level within 1 h after oral administration,^28^ which was 12-fold faster than the elimination of the cyclo­(Gly-Pro), which remained in serum for 12 h in our experiment. A study in humans has also reported that the *T*_max_, *C*_max_, and AUC_0–4h_ of Gly-Pro were lower than those of cyclo­(Gly-Pro).^29^ Similarly, the literature reports that Pro-Hyp dropped to basal levels within 3 h in mice,^28^ with an elimination rate faster than that of the CDP cyclo­(Pro-Hyp), which remained in the blood for 24 h in our experiment. These results were consistent with those in the FBMN analysis and highlighted the slower elimination rate of CDPs *in vivo* compared with their linear counterparts.

### 3.5 Neuroprotective Effects of CDPs Containing Pro/Hyp

#### 3.5.1 The CDPs Containing Pro/Hyp Increased the Cell Viability in Glu- and H_2_O_2_-Injured HT-22 Cells

Previous studies have demonstrated that CDPs exhibit diverse bioactivities, including anticancer, antimicrobial, neuroprotective, and anti-inflammatory effects.^9^ Twelve identified Hyp/Pro-CDPs exhibited prolonged systemic retention in murine circulation, making them potential substances for studying neuroprotective activity. Stimulation with excessive Glu and H_2_O_2_ can induce oxidative stress in HT22 cells and trigger DNA strand breaks and mitochondrial dysfunction, leading to cell death via ferroptosis and apoptosis. We initially explored the optimal compound concentration for inducing oxidative stress injury in HT22 cells. When exposed to 20 mM of Glu or 400 μM of H_2_O_2_ for 24 h, the cell viability was reduced to 40–60% (Figure S3A,B). Furthermore, these Hyp/Pro-CDPs significantly restored the loss of HT-22 cell viability caused by Glu at concentrations of 25 μM and 50 μM (*p* < 0.01, Table S11). The protective effect was highest at 50 μM of cyclo­(Hyp-Ala) (70.614 ± 4.85% cell viability, vs model group 40.759 ± 1.939% cell viability, *p* < 0.01; Figure 5A, Table S11), which is reported for the first time. The protective effect of cyclo­(Hyp-Gly), cyclo­(Pro-Ile), cyclo­(Pro-Phe), cyclo­(Hyp-Phe), and cyclo­(Hyp-Pro) on HT-22 cell viability reached more than 60% (63.703 ± 1.518, 63.104 ± 2.519, 61.107 ± 2.001, 60.206 ± 4.409, and 60.071 ± 3.101% cell viability, vs model group 40.759 ± 1.939% cell viability, *p* < 0.01; Figure 5A). At the same time, cyclo­(Hyp-Ala) also showed a good protective effect on HT-22 cell damage caused by H_2_O_2_, and the cell viability was up to 68.515 ± 2.290% at 50 μM (Figure 5B, Table S11). Other CDPs also showed neuroprotective effects in the H_2_O_2_-induced model (Figure 5B, Table S11). Notably, 50 μM of CDPs exhibited no detectable cytotoxicity to HT22 cells, demonstrating a favorable safety profile for neuronal applications (Figure S3C). These findings suggest that these CDPs may potentially protect neuronal cells from oxidation-induced damage to the neuronal cells.

**Figure 5:**
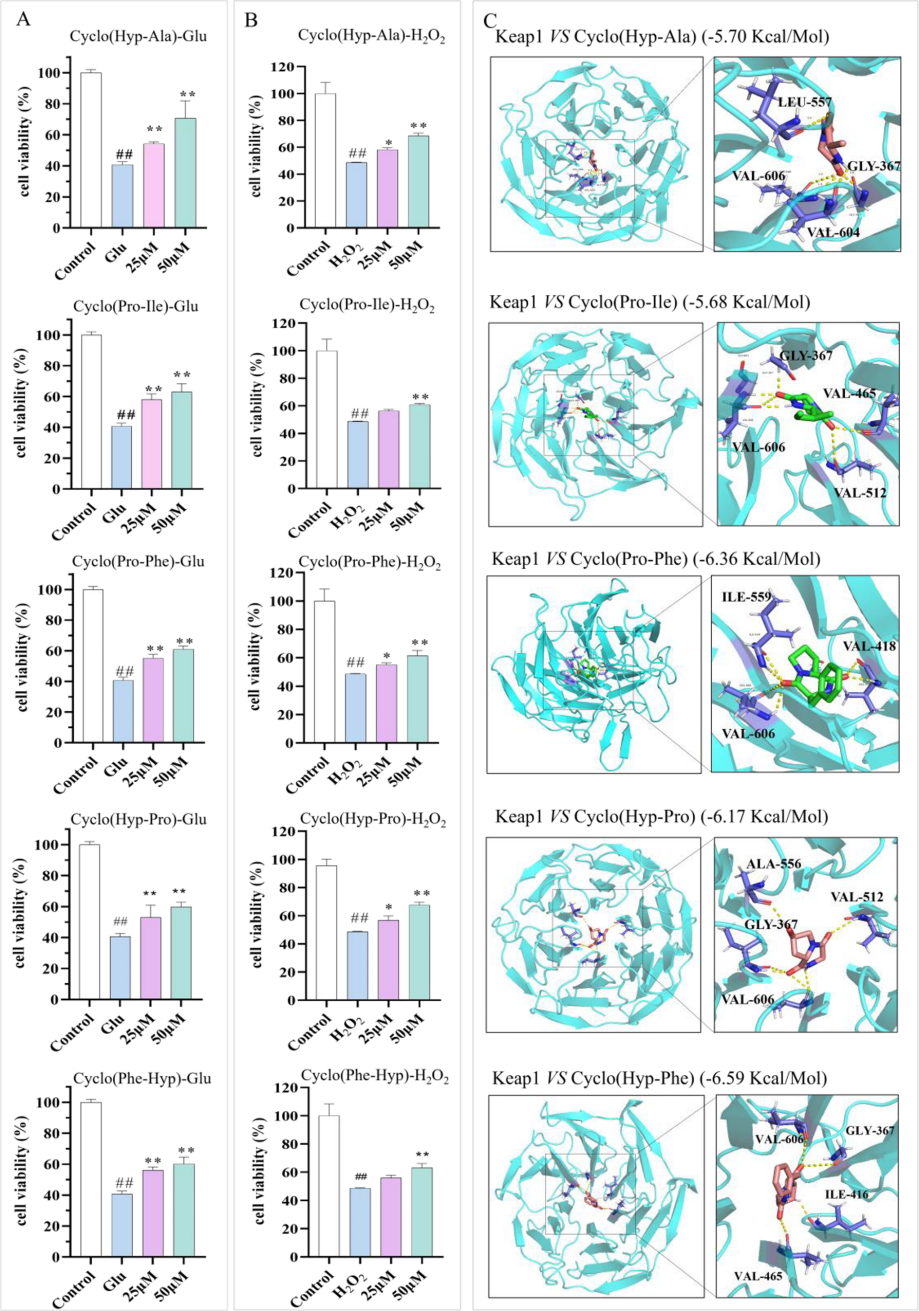
Neuroprotective activity of CDPs. (A) The CDPs increased the cell viability in Glu-injured HT-22 cells. (B) The CDPs increased the cell viability in H_2_O_2_-injured HT-22 cells. (C) Interaction of CDPs with keap-1 via molecular docking (vs model group: *p* < 0.05*, *p* < 0.01**; vs control group: *p* < 0.05^#^, *p* < 0.01^##^).

#### 3.5.2 Prediction of the Neuroprotective Mechanism of CDPs Using Molecular Docking

The above results indicated that these CDPs could protect HT-22 cells from Glu- and H_2_O_2_-induced oxidative stress. To further investigate their possible mechanisms, we performed a molecular docking simulation. Keap-1 is an important chaperone for E3 ubiquitin ligases.^30^ The transcription factor nuclear factor erythroid 2-related factor 2 (Nrf2) is redox potential-sensitive, activating antioxidative proteins to reduce reactive oxygen species (ROS).^31^ Inhibiting Keap-1 prevents Nrf2 degradation by the ubiquitin-proteasome system, leading to Nrf2 accumulation and its translocation to the nucleus, activating cellular defense mechanisms. Studies have shown that some small molecules can modify Keap-1 and impact the interaction of Keap-1 and Nrf2.^32^ To investigate the interactions of CDPs with the active site of Keap-1, we performed a molecular docking simulation. The top-ranked poses with the lowest binding energy were chosen, and their interactions with the corresponding amino acid residues were investigated. The calculated binding free energies for the CDPs ranged from −5 to −7 kcal/mol (as detailed in Table S11), indicating favorable interactions with the keap-1 protein. The docked poses of cyclo­(Hyp-Ala), cyclo­(Pro-Ile), cyclo­(Pro-Phe), cyclo­(Hyp-Phe), and cyclo­(Hyp-Pro) against Keap-1 are represented in Figure 5C, highlighting their spatial orientation and interaction profiles. It was distinguished that cyclo­(Hyp-Ala) fitted well into the active site via forming alkyl interaction and hydrogen bonds with critical amino acid residues, namely, Leu-557, Val-604, Val-606, and Gly-367. The distances of these bonds were measured at 3.4, 1.9, 3.5, 2.0, and 2.2 Å, respectively, suggesting a robust interaction that likely contributes to the stability of the cyclo­(Hyp-Ala) binding. The binding site of cyclic (Pro-Ile) consisted of Val-606, Val-512, Val-465, and Gly-367, with a binding energy of −5.68 kcal/mol. The free binding energy of cyclo­(Pro-Phe) was −6.36 kcal/mol, forming alkyl interactions and hydrogen bonds with Ile-559, Val-418, and Val-606. Furthermore, the binding sites for cyclo­(Hyp-Phe) were Val-606, Gly-367, Ile-416, and Val-465, with a binding energy of −6.59 kcal/mol. These results suggested that the CDPs may exert neuroprotective effects by binding to Keap-1, a well-recognized regulator of the Nrf2/ARE antioxidant pathway.^33,34^ Although molecular docking alone cannot confirm the biological mechanism, this target was selected based on its established role in oxidative stress defense. Nevertheless, further functional assays, such as Nrf2/ARE activation experiments, will be required to validate this pathway and clarify whether the observed binding translates into *in vivo* neuroprotective activity.^35,36^

In conclusion, the identification of *in vivo* bioactives of food gelatin is challenging due to the interference of endogenous proteins and peptides. In this study, we combined peptidomics with FBMN analysis and targeted spatiotemporal metabolomics to quickly and efficiently identify bioactive substances derived from the gelatin of donkey hides in the complex *in vivo* matrix. First, the peptidomics analysis results showed that the number of peptides detected in the small intestine decreased over time, and their size progressively became smaller. This suggests a progressive breakdown of the DHG protein into smaller peptides, a pattern commonly observed in protein digestion. The visualization capabilities of FBMN enabled us to rapidly identify CDPs from large data sets, marking the first report of CDPs being discovered in food gelatin. Furthermore, a targeted metabolomics approach was established and applied to find more CDPs, and we identified 35 CDPs. Among them, 12 Pro/Hyp-CDPs exhibited high abundance. The cell experiments demonstrated the neuroprotective activity of Hyp/Pro-CDPs, suggesting their potential critical role in the bioactivity of DHG; however, further verification of their mechanism of action is still required. This study highlights an effective strategy for identifying and characterizing exogenous bioactive substances in complex biological matrices, and the methodology can be extended to other food proteins, providing new avenues for exploring protein digestion processes and discovering bioactive compounds with potential health benefits. In addition, future studies integrating pathway-specific assays, microbiota-controlled models, and human-relevant digestion or clinical validation will be needed to strengthen the translational relevance.

## Supplementary Material





## Notes

FBMN results of peptides in the small intestine (Figure S1); MS/MS spectra of cyclic dipeptides in positive mode (Figure S2); Cytotoxicity studies of Glu, H_2_O_2_, and cyclic dipeptides on HT-22 cells: (A) cell survival after 24 h Glu induction; (B) cell survival after 24 h H_2_O_2_ induction; (C) cell survival after 24 h treatment with cyclic dipeptides (50 μM) (Figure S3) (PDF)
Identification of peptides in small intestinal contents at different time points after DHG administration (0.5, 1, 2, 4, 8, and 12 h) in Tables S1–S6; The peptides smaller than 690 Da in the small intestine (Table S7); Cyclic dipeptide database (Table S8); Summary of cyclic dipeptides in serum (Table S9); Linearity and sensitivity for CDPs quantitation in DHG and serum (Table S10); Neuroprotective activity of cyclic dipeptides (Table S11) (XLSX)
