# Supplementary material for: In Vivo Atlas of Neuroprotective Cyclic Dipeptides Derived from Food Gelatin Using Peptidomics and Feature-Based Molecular Networking
Source: J Agric Food Chem. 2025 Oct 22;73(45):28811–22. doi: 10.1021/acs.jafc.5c08112 (PMC12616683; doi:10.1021/acs.jafc.5c08112)
Supplement: Supplementary file 1 [file jf5c08112_si_001.pdf]

Food gelatin-derived neuroprotective cyclic dipeptides atlas *in vivo* using peptidomics and feature-based molecular networking

Pingping Dong[1], Lanjia Ao[1], Yujie Li[1], Haoyuan Zeng[1], Yanmin Zhang[3], Haibo Zou[2], Jing Leng[4], Na Li[1*], Jian-Lin Wu[1*]

Figure S1. FBMN results of peptides in small intestine.

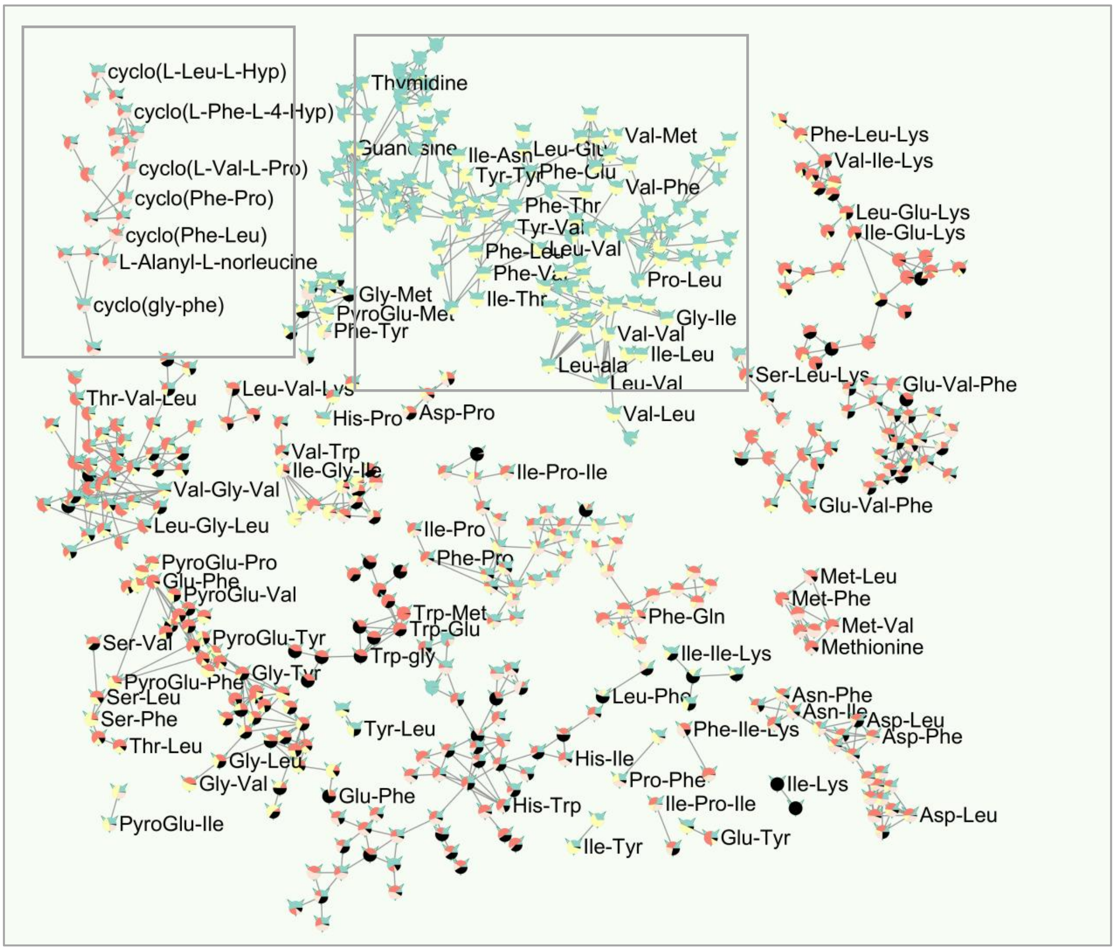

Figure S2. The MS/MS spectra of cyclic dipeptides in positive mode.

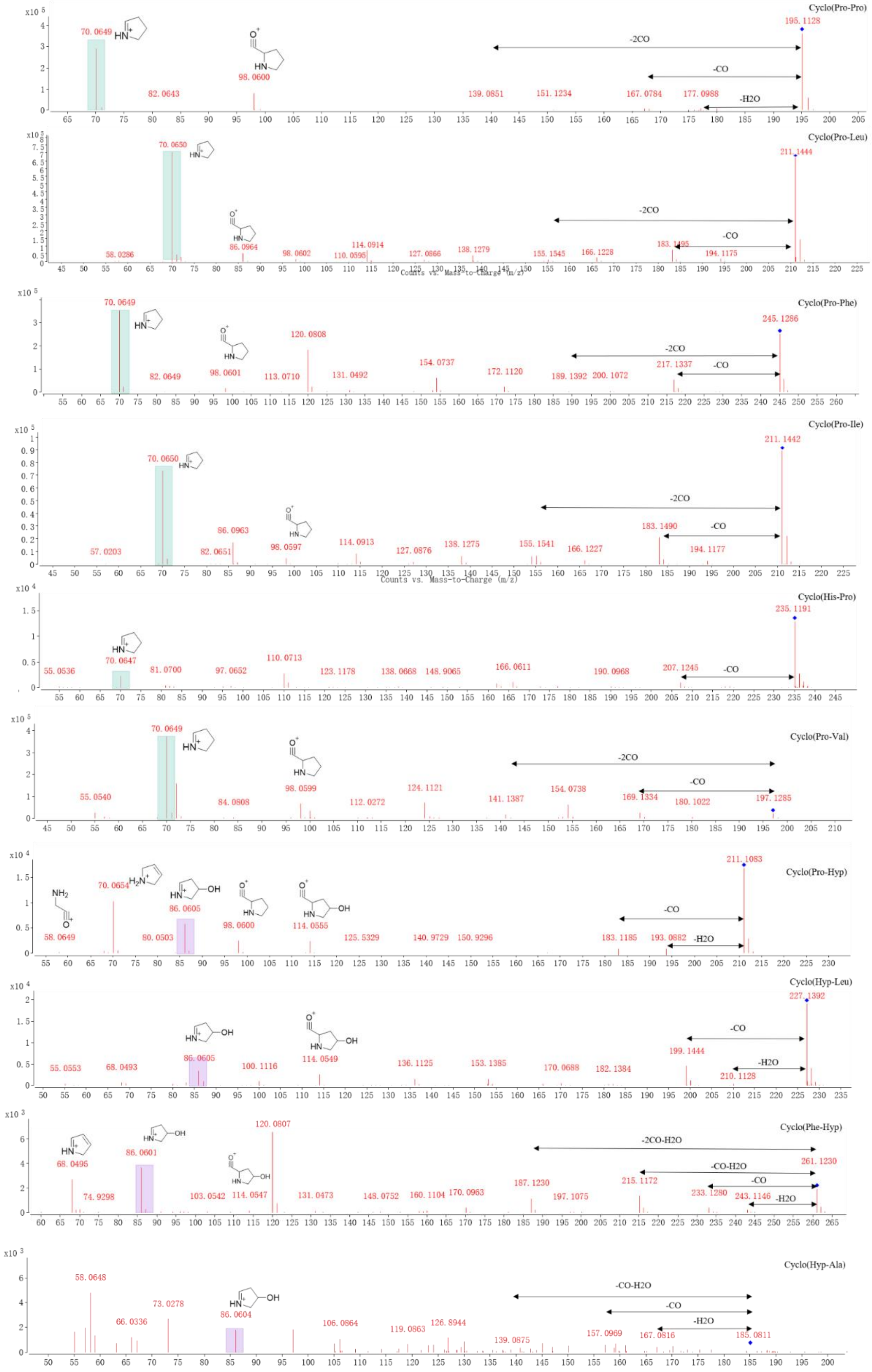

Figure S3. Study on the cytotoxicity of Glu, H_2O_2 and cyclic dipeptide on HT-22 cells.

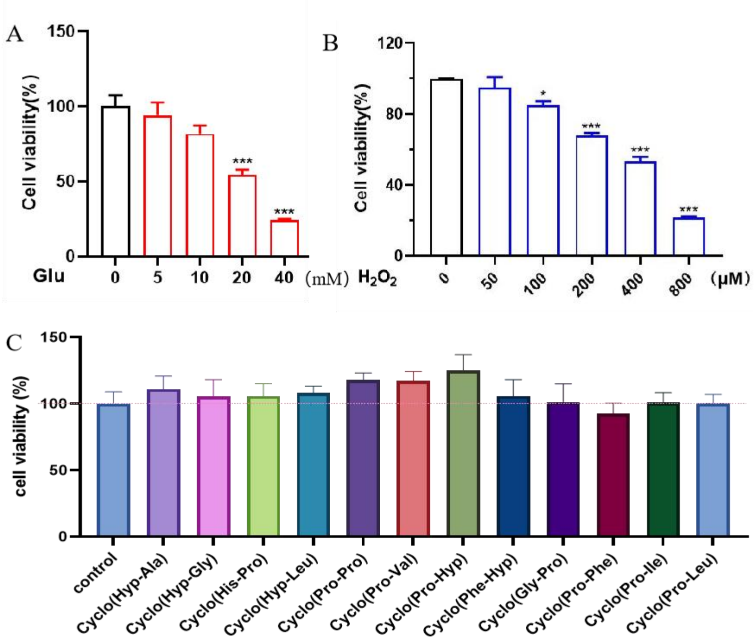

(**A**) HT-22 cell survival rate after Glu induction for 24 h. (**B**) HT-22 cell survival rate after H_2O_2 induction for 24 h. (**C**) HT-22 cell survival rate after 24 h administration of each cyclic dipeptide (50 uM).
